# A Protocol for Remote Cognitive Training Developed for Use in Clinical Populations During the COVID-19 Pandemic

**DOI:** 10.1089/neur.2023.0009

**Published:** 2023-08-14

**Authors:** Taylor Snowden, Lisa Ohlhauser, Jamie Morrison, Jocelyn Faubert, Jodie Gawryluk, Brian R. Christie

**Affiliations:** ^1^ Division of Medical Sciences, University of Victoria, Victoria, British Columbia, Canada.; ^2^ Institute of Aging and Lifelong Health, University of Victoria, Victoria, British Columbia, Canada.; ^3^ Department of Psychology, University of Victoria, Victoria, British Columbia, Canada.; ^4^ Université de Montréal, Montréal, Quebec, Canada.; ^5^ Island Medical Program, University of British Columbia, Victoria, British Columbia, Canada.

**Keywords:** 3D-MOT, cognitive training, healthy aging, NeuroTrackerX, three-dimensional multiple object tracking

## Abstract

Many traumatic brain injury (TBI) survivors face scheduling and transportation challenges when seeking therapeutic interventions. The COVID-19 pandemic created a shift in the use of at-home spaces for work, play, and research, inspiring the development of online therapeutic options. In the current study, we determined the feasibility of an at-home cognitive training tool (NeuroTrackerX) that uses anaglyph three-dimensional (3D) glasses and three-dimensional multiple object tracking (3D-MOT) software. We recruited 20 adults (10 female; mean age = 68.3 years, standard deviation [SD] = 6.75) as the at-home training group. We assessed cognitive health status for participants using a self-report questionnaire and the Mini-Mental State Examination (MMSE), and all participants were deemed cognitively healthy (MMSE >26). At-home participants loaned the necessary equipment (e.g., 3D glasses, computer equipment) from the research facilities and engaged in 10 training sessions over 5 weeks (two times per week). Participant recruitment, retention, adherence, and experience were used as markers of feasibility. For program validation, 20 participants (10 female; mean age = 63.39 years, SD = 12.22), who had previously completed at least eight sessions of the in-lab 3D-MOT program, were randomly selected as the control group. We assessed individual session scores, overall improvement, and learning rates between groups. Program feasibility is supported by high recruitment and retention, 90% participant adherence, and participants' ease of use of the program. Validation of the program is supported. Groups showed no differences in session scores (*p* > 0.05) and percentage improvement (*p* > 0.05) despite the differences in screen size and 3D technology. Participants in both groups showed significant improvements in task performance across the training sessions (*p* < 0.001). NeuroTrackerX provides a promising at-home option for cognitive training in cognitively healthy adults and may be a promising avenue as an at-home therapeutic for TBI survivors. This abstract was previously published on clinicaltrials.gov and can be found at: https://www.clinicaltrials.gov/ct2/show/NCT05278273

## Introduction

The COVID-19 pandemic has created a shift in the use of at-home spaces for work, play, and research. Since the start of 2020, clinical research populations have been limited, and research teams worldwide have adapted their investigative techniques. At-home interventions, where one can participate regardless of the outside environment, appear to be an encouraging avenue for research moving forward. One advantage of at-home interventions is the potential to serve populations that typically cannot partake in clinical trials because of lack of accessibility, as can be the case for brain injury survivors, who are disproportionately affected by barriers to access such as difficulties with driving or public transport.^1,2^ However, at-home research has complications that should be addressed before population-focused research. This includes assessing the feasibility of the method and validating the program to address the potential for reduced standardization attributable to the differences between the in-lab and at-home versions of the program.

Enhancing cognitive function is a topic of significant interest for many groups, including brain injury survivors and aging adults. Several cognitive enhancement interventions to date have included puzzle games (e.g., Sudoku),^3^ music therapy,^4^ and healthy-lifestyle interventions.^5^ Computer-based cognitive training is another attractive method because of its accessibility and ease of use. Unfortunately, though computerized cognitive tasks have shown cognitive benefits similar to more traditional methods over the short term, the transfer effect to real life has remained modest for most training tools currently on the market.^6,7^

Three-dimensional multiple object tracking (3D-MOT) is a type of cognitive training that requires integrating multiple cognitive networks, including attention, memory, and processing power. This type of cognitive training tool creates a complex and dynamic virtual three-dimensional (3D) environment that can be highly translatable to real-world situations.^8–10^ Specifically, using the NeuroTracker software, researchers observe cognitive improvements in population samples that include healthy young adults,^11^ healthy older adults,^12^ and older adults with subjective memory decline.^13^ Ongoing research suggests that 3D-MOT is also a promising therapeutic and cognitive training tool for brain injury survivors.^14,15^ In a push for better telehealth options, the manufacturer of NeuroTracker released an at-home version of the cognitive training tool called NeuroTrackerX. The manufacturer suggests that NeuroTrackerX offers the same benefits as the traditional version; however, it has not yet been tested for clinical research.

Key aspects of NeuroTracker training for research include having participants perform the task within standardized lab settings (i.e., lab assistants present, using active 3D technology, and performing NeuroTracker on a wide screen to encourage the use of peripheral visual strategies during the task). These key aspects are modified for the at-home version, specifically anaglyph 3D glasses and a smaller screen size. Therefore, we undertook the current study to assess the feasibility of this new tool for research purposes by assessing participant recruitment, retention, adherence, and experience. Further, we examined the validity of the program by comparing results from the in-lab program to the at-home program.

## Methods

### Participants

We recruited 20 cognitively healthy adults through local retiree associations and word of mouth. To serve as a control group for the validation component, we randomly selected 20 cognitively healthy participants (10 male, 10 female) who had previously completed at least eight sessions of the in-lab version of NeuroTracker. Inclusion criteria for both groups included being ≥50 years of age and to have either normal or corrected vision. Exclusion criteria included the presence of any major neurocognitive disorder or the presence of visual deficits that impeded one's ability to complete the 3D task. In addition to the self-report of cognitive status, all at-home and 15 of 20 in-lab participants completed the Mini-Mental State Examination (MMSE) to measure cognitive status before NeuroTracker training. Because the in-lab group was randomly selected from previous participants, not all subjects had completed the MMSE.

### Data acquisition

We collected data from the online NeuroTrackerX user-researcher interface, which provided access to at-home participant data. In one circumstance, a computer error occurred that affected the NeuroTrackerX program. This produced an incomplete score that was removed from that session, and the participant's average score of the trials immediately before and after was imputed. The doctoral-level clinical neuropsychology student (L.O.) administered and scored at-home MMSEs using a secure online video platform (i.e., Zoom). For any tasks involving drawing, participants were instructed to adjust the camera such that their hands and paper were in view. MMSE scores >26 were used to rule out the likelihood of dementia, and in combination with the participants' self-reports, participants were deemed cognitively healthy.

### Three-dimensional multiple object tracking with NeuroTracker software

**FIG. 1.**
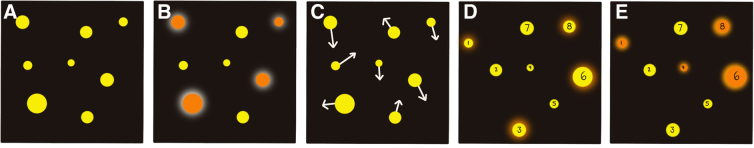
Depiction of the five stages of a NeuroTracker Core mode trial. (**A**) Eight yellow balls are presented in 3D space. (**B**) Four targets are presented in orange with a white halo. (**C**) All balls go back to yellow and move in random directions for 8 sec. The goal of the task is to track the original targets throughout the moving period. (**D**) Balls stop moving and are numerically labeled. The participant identifies the original four targets using the mouse or corresponding keys. Selected balls have an orange halo. (**E**) Feedback is provided. The correct targets are identified in orange with an orange halo. The cycle then restarts and continues for the remaining trials. The speed of the balls is adjusted based on the success of the previous trial. Completion of 20 trials completes one session. 3D, three-dimensional.

The experimental procedure for both versions (in-lab vs. at-home) is similar, and the premise of the task remains the same. All sessions followed the pre-programmed Core mode, and one session consists of three blocks of 20 trials. One trial consists of five stages (Fig. 1). All Core trials had four target balls and four distractor balls. If the participant correctly identifies all the target balls at the end of a trial, the speed increases by 0.05 log units. If the participant misses any balls, the speed is decreased by 0.05 log units, making this task highly adaptable to a person's ability. At the end of 20 trials, the program presents the participant's score. The score is called a speed threshold and has arbitrary units. The speed threshold represents the speed at which a participant can successfully track the balls 50% of the time and indicates the speed at which performance begins to break down. For reference, a speed threshold of 1.0 equates to a tracking speed of 68 cm/s; higher speed thresholds indicate better tracking abilities and performance in general.

### In-lab training

**FIG. 2.**
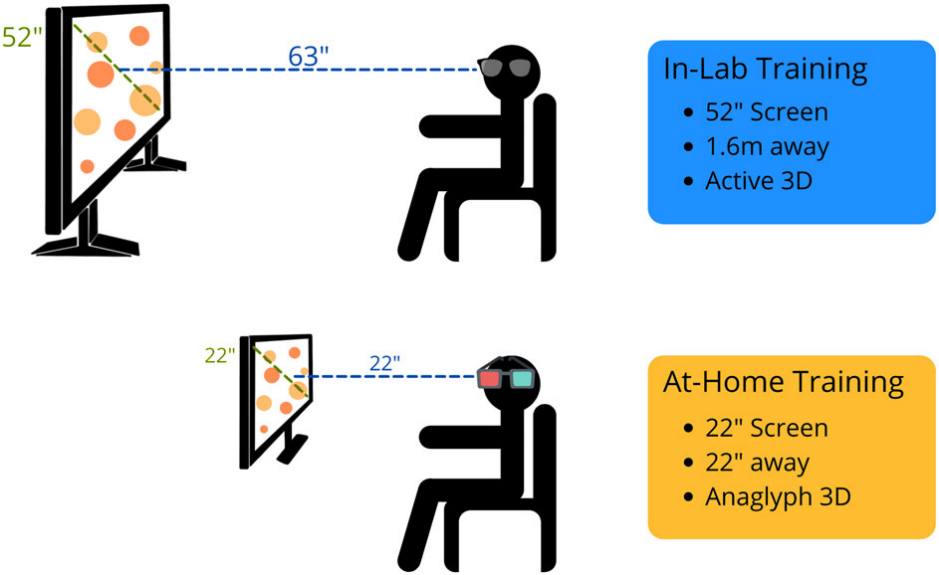
Depiction of the in-lab and at-home versions of NeuroTracker. The main differences are screen size, distance from the screen, and the technology used to allow 3D perception (active vs. anaglyph). 3D, three-dimensional.

For the in-lab training, the setup is the same as previously described.^16^ Briefly, the research assistant instructed the participant to sit upright, 1.6 m away from the screen. The 52-inch Samsung screen used was compatible with active 3D technology, and participants wore the provided Samsung active 3D glasses (Fig. 2). Participants could also request noise-cancelling headphones to wear during the sessions. Before the first session, the research assistant read the standard instructions to the participant.^17^ The participant manually entered all of their answers, and to better ensure understanding of the task, the research assistant stayed in the training room until the participant completed their first successful trial.

### At-home training

To improve standardization and accessibility of the at-home training process, our lab provided computers, 22-inch diagonal-width computer monitors, NeuroTracker anaglyph 3D glasses, and noise-cancelling headphones to participants, if needed (Fig. 2). In addition, the research team provided a digital set-up manual to participants, which included the standard NeuroTracker instructions (see Supplementary File S1). Participants were encouraged to sit comfortably in a quiet environment. As recommended by the NeuroTrackerX team, for the optimum anaglyph 3D experience, participants were advised to sit at a distance from the monitor equal to the diagonal width of the computer (i.e., for a 22-inch monitor, the participant would sit 22 inch away from the screen). The research team sent weekly e-mails to the participants to check in. If participants were experiencing any challenges, the research team provided phone- and/or video-call check-ins to assist.

### Experimental timeline

**FIG. 3.**
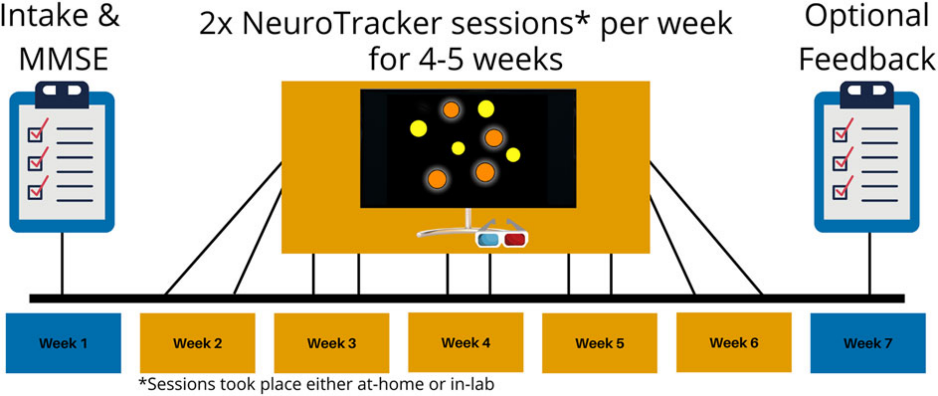
Generalized experimental timeline for the in-lab and at-home training groups. Both groups underwent an intake session to collect demographic information and complete the Mini-Mental State Examination (MMSE). Then, the groups underwent NeuroTracker training at home, or in the lab, two times per week for 4–5 weeks. Some participants in the in-lab group only completed 4 weeks of NeuroTracker training because of lab closures during the COVID-19 pandemic. Participants in the at-home group had an optional session to provide feedback about their experience with the at-home NeuroTracker program.

All participants followed the same experimental timeline (Fig. 3). Participants completed 60 individual NeuroTracker trials per day, with the sessions presented as three blocks of 20 trials (Fig. 2). NeuroTracker sessions were completed twice per week for 4–5 weeks, totaling 8–10 completed sessions (460–600 trials total). Six of the randomly selected in-lab participants completed 4 of 5 weeks of training because of laboratory closures during the COVID-19 pandemic.

### Feasibility

Feasibility of the at-home program was assessed using raw participant recruitment numbers, participant retention (ongoing participation), and adherence (compliance with the prescribed intervention). Participants in the at-home training group also had the opportunity to provide optional feedback about their experience with the program. This survey included Likert-scale–based questions and open-ended responses.

### Statistical analysis

For this experimental, non-random design, we assessed between-group demographics using Student's *t*-tests and report test statistics, *p* values, and 95% confidence intervals (CIs). Further, we quantified learning-related changes within the task itself for each participant by averaging the three speed thresholds (NeuroTracker/NeuroTrackerX scores) from each training day. Estimation statistics (e.g., CIs, effect sizes) provide a means to examine data without relying solely on *p* values.^18,19^ We assessed between-group differences in NeuroTracker scores for each session using estimation statistics and present the effect size as mean differences (in-lab minus at-home) with 95% CIs using 5000 bootstrap samples. The CI interval is bias-corrected and accelerated. We also present *p* values as representations of the likelihood of observing the effect size if the null hypothesis of zero difference is true. For each permutation *p* value, the program performed 5000 reshuffles of the at-home and in-lab labels.

Additionally, to assess for group differences in performance across time, we applied a longitudinal multi-level conditional growth model with a fixed slope. All models used maximum likelihood estimation. Percentage improvement was calculated by subtracting a participant's average baseline score from their final score, dividing by the baseline score, and multiplying by 100 (see equation below). We used the dabestr^20^ package in R Studio (version 1.3.1093) to generate all estimation statistics and plots.

$$\begin{array}{cc} & PercentageImprovement=\\ & \frac{Avg.FinalScore-Avg.BaselineScore}{Avg.BaselineScore}\times 100\% \end{array}$$

## Results

### Demographics

There was no statistically significant age difference between the at-home and in-lab training groups (*t*_[28]_ = 1.56, *p* = 0.1291; 95% CI: −1.57, 11.74). Further, there were no statistically significant differences between MMSE scores (*t*_[22]_ = 1.14, *p* = 0.2674; 95% CI: −0.054, 1.84), and all participants self-reported as being cognitively healthy (i.e., no diagnosis of a neurodegenerative disorder). There were equal numbers of males and females in each group (10 females per group). Two participants (1 male, 1 female) did not complete the sessions as instructed and were excluded from the analysis. One participant took a 3-week break from sessions because of an unrelated injury and subsequent surgery, completing four sessions total. The other participant only completed one training day per week (five sessions total) because of time constraints. Participant demographics are available in Table 1.

**Table 1.** Participant Demographics of Those Included in the Final Analysis

	At-home mean (SD, range)	In-lab mean (SD, range)
Age	68.39 (6.75, 57–83)	63.39 (12.22, 50–87)
MMSE	29.22 (1.26, 27–30)	28.57 (1.26, 26–30)
Sex	9 male, 9 female	10 male, 10 female

Means and standard deviations were calculated for age and MMSE scores. The number of males and females is also included.

MMSE, Mini-Mental State Examination; SD, standard deviation.

### Feasibility of the at-home program

Feasibility of the at-home program was assessed through participant recruitment, retention, and adherence, as well as participant experience with the program. Our recruitment goal was to recruit 20 participants in 14 days. Our recruitment expectations were exceeded. Within the 2-week period, we received 32 responses. Because of limited equipment, we randomly selected 20 participants for participation in the study. Retention was defined as the percentage of participants who stayed in the study for the 5-week time period, and the retention rate was 100%. Participant adherence was defined as the percentage of participants who completed the intervention as described (i.e., two sessions per week for 5 weeks) and was found to be 90%. After the study, participants were invited to rate their experience with the program on a Likert-based scale. Fifteen participants offered feedback on the following questions (Tables 2–4): 1) How would you rate NeuroTrackerX's ease of use?; 2) How would you rate the clarity of the provided instructions?; and 3) How feasible was adhering to this intervention?

**Table 2.** Participant-Rated Ease of Use of the Program

	Extremely difficult	Difficult	Neutral	Easy	Extremely easy
No. of responses (*n*; %)	0; 0	0; 0	0; 0	9; 60	6; 40

Participant responses to the following question, “On the following scale, please rate NeuroTrackerX's ease of use.”

**Table 3.** Participant-Rated Clarity of the Provided Instructions

	Extremely unclear	Unclear	Neutral	Clear	Extremely clear
No. of responses (*n*; %)	0; 0	0; 0	1; 6	5; 33	9; 60

Participant responses to the following question, “On the following scale, please rate the clarity of the provided instructions.” The provided instructions are included in Supplementary File S1.

**Table 4.** Participant-Rated Feasibility of the Intervention

	Extremely unfeasible	Unfeasible	Neutral	Feasible	Extremely feasible
No. of responses (*n*; %)	0; 0	0; 0	2; 13	7; 46	6; 40

Participant responses to the following question, “How would you rate the feasibility of adhering to this intervention (i.e., sticking to training twice per week for five weeks).”

### Program validation: No between-group differences in sessional scores

**FIG. 4.**
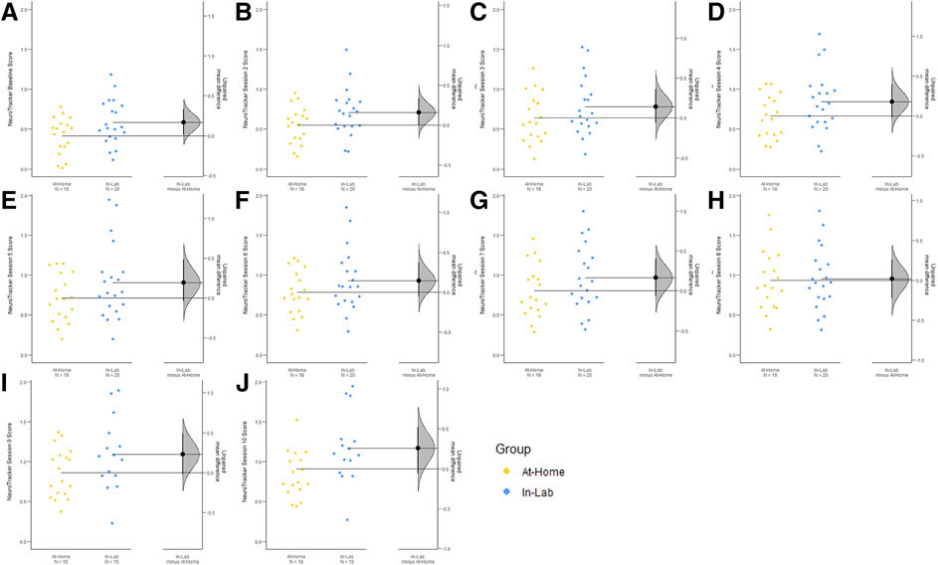
Mean difference in session score between in-lab (yellow) and at-home (blue) groups are shown in the above Gardner-Altman estimation plots. Sessions 1 (baseline) through 10 are depicted by (A) to (J), respectively. Both groups are plotted on the left axes; the mean difference is plotted on a floating axis on the right as a bootstrap sampling distribution (5000 samples). Unpaired mean difference is depicted as a dot (black); the 95% confidence interval is indicated by the ends of the vertical error bar.

There were no differences between in-lab and at-home groups at any session. However, there was a trend toward a statistically significant difference between the two groups in the baseline session (unpaired mean difference = 0.0752; 95% CI: −0.0627, 0.216; *p* = 0.05356; Fig. 4A), indicating that the in-lab group trended toward a better performance on the baseline session. Details regarding the specific data, results, and analyses used can be found in Table 5.

**Table 5.** Session Differences Between At-Home and In-Lab Participants

Session	Data structure	Mean score (at-home; in-lab)	Mean difference [95% CI]	Test value;* p *value (Student's* t*-test)	Interpretation
1 (Baseline)	Normal distribution	0.42; 0.58	0.0752 [−0.0627, 0.216]	*t*_[35]_ = 1.996; *p =* 0.05356	No difference; trending In-lab > at-home
2	Normal distribution	0.55; 0.71	0.161 [0.00273; 0.337]	*t*_[35]_ = 1.853; *p =* 0.07225	No difference
3	Normal distribution	0.64; 0.78	0.143 [0.0627; 0.363]	*t*_[35]_ = 1.310; *p =* 0.1984	No difference
4	Normal distribution	0.66; 0.84	0.179 [0.0169; 0.393]	*t*_[34]_ = 1.692; *p =* 0.0998	No difference
5	Normal distribution	0.71; 0.91	0.194 [0.0365; 0.478]	*t*_[32]_ = 1.528; *p =* 0.1363	No difference
6	Normal distribution	0.79; 0.93	0.144 [0.0571; 0.368]	*t*_[33]_ = 1.5275; *p =* 0.1892	No difference
7	Normal distribution	0.80; 0.96	0.163 [0.0707; 0.398]	*t*_[35]_ = 1.340; *p =* 0.1872	No difference
8	Normal distribution	0.93; 0.96	0.0183 [0.229; 0.259]	*t*_[35]_ = 0.147; *p =* 0.8839	No difference
9	Normal distribution	0.86; 1.09	0.173 [0.077; 0.419]	*t*_[23]_ = 1.672; *p =* 0.1076	No difference
10	Normal distribution	0.91; 1.05	0.202 [0.108; 0.458]	*t*_[29]_ = 1.706; *p =* 0.0986	No difference

Mean difference was calculated as in-lab score minus at-home score with 95% confidence intervals (CIs). CIs were calculated using 5000 bootstrap samples and are bias-corrected and accelerated. For each permutation *p* value, 5000 reshuffles of the at-home and in-lab labels were performed. Higher scores indicate better performance.

### Program validation: No between-group differences in performance across time

First, an unconditional means model—with participant ID as the nested variable and random effect and NeuroTracker score as the outcome variable—was applied to the data (Supplementary Table S1) to determine how much the grand mean of the intercept differs from zero. In the unconditional means model, the intercept was determined to be 0.801, which significantly differed from zero. The intraclass correlation coefficient (ICC) was found to be 0.6528 indicating that clustering is taking place and to move forward with additional models.

An unconditional growth model with a fixed slope was then applied. Participant ID was the nested variable and random effect, and session was included as a fixed effect. NeuroTracker score was the outcome variable. A fixed slope was chosen because participants generally showed improvement in NeuroTracker score over time, and having a fixed slope captured the fit of the model as evidenced by significant *p* values at each session (Supplementary Table S2). Deviance statistics were used to compare the unconditional means model to the unconditional growth model. The logLik value was significantly increased in the unconditional growth model (L. ratio = 205.6, *p* < 0.0001), indicating that the unconditional growth model is a better fit. The ICC was found to be 0.777, indicating that this model accounts for a greater amount of variability of clustering.

**FIG. 5.**
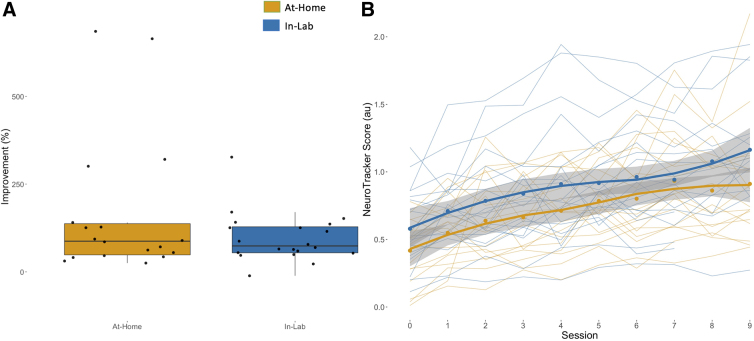
NeuroTracker score improvement across sessions. (**A**) A box plot showing percentage improvement from the first to the final session. Participants' improvements are plotted as black dots within their respective group (yellow, at-home; blue, in-lab). There was no between-group difference in percentage improvement (*p* = 0.1035; 95% CI: −286.81, 28.9). (**B**) Individual participant scores are shown as thin, connected lines. Group mean scores by session are shown as dots with thicker, smoothened lines, surrounded by 95% confidence intervals (CI; gray).

To test whether group (at-home or in-lab) affected NeuroTracker scores over time, a conditional growth model with a fixed slope was applied (Supplementary Table S3). The model was the same as the unconditional growth model, but group was included as a predictor. Interactions between session and group were also tested, but no statistically significant interactions were observed. Group was not a significant predictor of NeuroTracker score over time (*p* = 0.1489), indicating no differences between the groups across sessions (Fig. 5B). Deviance statistics were used to compare the unconditional growth model to the conditional growth model (i.e., does group influence NeuroTracker scores over time). Including group as a predictor did not significantly change the logLik value of the model (L. ratio = 2.17, *p* = 0.14), suggesting that there is no difference between the models. The proportion reduction of the conditional growth model was calculated to determine how much variability was being explained by including group as a predictor. The proportion reduction between the unconditional growth model and the conditional growth model was 0.057, indicating that group only accounted for 5.7% of the variance captured in the full model.

### Equivalent improvement in NeuroTracker score from first to final session

There was no between-group difference in percentage improvement (*p* = 0.1035; 95% CI: −286.81, 28.9; Fig. 5A). Percentage improvement was included to control for the participant's baseline score. Median percentage improvement in the in-lab group was 73.7%, with a mean value of 96.6%. Median percentage improvement in the at-home group was 79.4%, with a mean value of 163.8%. The paired mean difference between the at-home group's first and final NeuroTracker score was 0.494 (95% CI: 0.315, 0.761; *p* = 0.0000747). The paired mean difference between the in-lab group's first and final session was 0.463 (95% CI: 0.237, 0.694; *p* = 0.000174). Full results can be found in Supplementary Table S4. Both groups showed improvement on the task, and each group's learning curves exhibited similar trajectories (Fig. 5B).

## Discussion

The general purpose of this study was to test the feasibility and describe a protocol for at-home cognitive training using NeuroTrackerX in cognitively healthy adults for research purposes. The present study describes a feasible study design given the participant interest, high adherence, and the participant-identified ease and feasibility of the program. A secondary aim of this study was to validate NeuroTrackerX for research purposes. We compared the performance of cognitively healthy adults (based on an MMSE >26 and self-report) on the at-home and in-lab versions over 8–10 sessions (480–600 trials). We observed no differences in performance between groups on any of the sessions; however, there did appear to be a subtle difference between groups on the first session, which can be observed in Figure 4A. Further, both groups had similar improvements throughout the sessions, and group was not a significant predictor of NeuroTracker scores across time.

We suspect that the subtle first-session differences may be attributed to environmental differences between the at-home and in-lab groups. For example, when a participant comes to the lab for the first time, the research assistant explains the task to the participants and allows the participant to ask any questions before starting. The research assistant also stays in the same room as the participant during the first few trials to ensure that they understand the task and that the program is running correctly. Although the research assistant is in the room, they do not provide any guidance on the correct answers. In the at-home program, participants were given an instruction manual (Supplementary File S1), and no live instructions were provided. After completing the at-home sessions, participants had the opportunity to complete an exit interview to share their experience with the at-home program. Fifteen participants offered feedback on the program. Though all participants rated the program as “easy to use” or “extremely easy to use,” 7 participants disclosed that they felt confused on the first session, even after reading the provided instructions. This confusion may have contributed to the slightly lower scores on the baseline session in the at-home group. Five participants chose not to complete the optional exit interview; however, information about their reason for not providing feedback was not collected.

There were no between-group differences in overall percentage improvement. The at-home group showed a median percentage improvement of 79.4% whereas the in-lab group showed a median percentage improvement of 73.7%. Additionally, the at-home group improved an average of 0.49 points, whereas the in-lab group improved an average of 0.46 points. These results are consistent with previous MSc thesis research demonstrating improvements in NeuroTracker in adults ≥61 years of age, improving an average of 0.54 points over 10 sessions (Shaw, 2019). Further, longitudinal multi-level modeling demonstrated no difference in performance trajectory between groups, and average scores from both groups followed a logarithmic progression, as observed in previous research.^12,13^

Altogether, our results suggest that the at-home version of NeuroTracker offers the same experience to participants as the in-lab version. Future researchers using NeuroTrackerX as a cognitive training tool should follow the steps outlined in this article for standardization of practice. This study was the first step in moving toward a more accessible future for cognitive training, specifically using the NeuroTrackerX platform.

### Limitations and suggestions for future research

It is important to acknowledge limitations of the current study. First, the control group was not directly recruited for this study; therefore, randomized assignment was not possible. Given the ongoing COVID-19 pandemic, in-person research was not possible, so we chose to include *post hoc* analyses to compare the in-lab versus at-home programs. Although we matched participants based on age and sex, other factors are known to impact NeuroTracker performance (e.g., elite and amateur athletes^21^). Although we did not control for this in the present study, no participants had participated at an elite level in the past 20 years. Second, we were not able to include two at-home participants in the final analysis. One participant took an extended break during the intervention because of an unrelated injury, whereas the other participant only completed one session per week because of time constraints. Third, the official cognitive health of participants cannot be confirmed because we did not conduct a battery of neuropsychological tests. However, given the self-reports and the MMSE screening tool, we are confident that that no participants were in advanced cognitive decline. This study was sufficiently powered to observe medium effect sizes (*F* > 0.45); therefore, future studies should look to include a larger sample of participants. Despite these limitations, we are excited about future research areas for this program.

NeuroTracker software is used by researchers across the world across various fields of study. With the COVID-19 pandemic, many research labs were forced to shift to online and at-home platforms. This is the first study to assess NeuroTrackerX for research purposes. Based on the participant adherence, participant-identified program ease, and participant-identified intervention feasibility identified in the present study, we recommend that researchers can use NeuroTrackerX as part of their intervention protocols.

Based on exit-interview conversations, we propose to video conference with participants to go over the instruction manual and allow participants to ask any questions they may have before their first session. Further, 6 participants identified that having a “trial run” to learn the task would make the task easier.

Given the enormous scope of NeuroTracker research, from athletic enhancement^17,22,23^ to military performance^24^ and concussion detection,^16,25^ researchers worldwide can begin using NeuroTrackerX. Specifically, we suggest examining the feasibility of this tool for populations that could benefit from cognitive training but may have difficulty with transportation (e.g., older adults, persons with TBI), who have previously benefitted from the in-lab version. Although this study did not assess for transfer effects (i.e., cognitive changes after engaging in a NeuroTrackerX intervention), future studies should examine NeuroTrackerX as a cognitive training tool for the aforementioned populations.

## Conclusion

NeuroTrackerX is a feasible method of at-home cognitive training in cognitively healthy adults for research purposes. When using NeuroTrackerX Core mode for research purposes, we recommend standardizing the screen size and encouraging participants to train twice per week with 1- to 2-day intervals between sessions in a distraction-free environment. We also recommended that participants train during a consistent time of day, though this was not a requirement for the present study. Under these conditions, this research suggests that the at-home version, NeuroTrackerX, offers a very similar training environment as the in-lab version and can be used for research purposes.

## Supplementary Material

Supplemental data

Supplemental data

Supplemental data

Supplemental data

Supplemental data

## Data Availability

The data sets used and/or analyzed during the current study are available from the corresponding author on reasonable request.

## Abbreviations Used

3D: three-dimensional
3D-MOT: three-dimensional multiple object tracking
CIs: confidence intervals
ICC: intraclass correlation coefficient
MMSE: Mini-Mental State Examination
SD: standard deviation
TBI: traumatic brain injury

## References

[1] Dams-O'Connor K, Landau A, Hoffman J, et al. Patient perspectives on quality and access to healthcare after brain injury. Brain Inj 2018;32(4):431–441; doi: 10.1080/02699052.2018.1429024 29388840

[2] Fisk GD, Schneider JJ, Novack TA. Driving following traumatic brain injury: prevalence, exposure, advice and evaluations. Brain Inj 2009;12(8):683–695; doi: 10.1080/026990598122241 9724839

[3] Grabbe JW. Sudoku and working memory performance for older adults. Act Adapt Aging 2011;35(3):241–254; doi: 10.1080/01924788.2011.596748

[4] Román-Caballero R, Arnedo M, Triviño M, et al. Musical practice as an enhancer of cognitive function in healthy aging—a systematic review and meta-analysis. PLoS One 2018;13(11):e0207957; doi: 10.1371/journal.pone.0207957 30481227 PMC6258526

[5] Miller KJ, Siddarth P, Gaines JM, et al. The memory fitness program: cognitive effects of a healthy aging intervention. Am J Geriatr Psychiatry 2012;20(6):514–523; doi: 10.1097/JGP.0b013e318227f821 21765343 PMC4255461

[6] Sala G, Aksayli DN, Tatlidil SK, et al. Near and far transfer in cognitive training: a second-order meta-analysis. Collabra Psychol 2019;5(1):18; doi: 10.1525/collabra.203

[7] Kueider AM, Parisi JM, Gross AL, et al. Computerized cognitive training with older adults: a systematic review. PLoS One 2012;7(7):e40588; doi: 10.1371/journal.pone.0040588 22792378 PMC3394709

[8] Michaels J, Chaumillon R, Nguyen-Tri D, et al. Driving simulator scenarios and measures to faithfully evaluate risky driving behavior: a comparative study of different driver age groups. PLoS One 2017;12(10):e0185909; doi: 10.1371/journal.pone.0185909 29016693 PMC5634611

[9] Legault I, Faubert J. Perceptual-cognitive training improves biological motion perception: evidence for transferability of training in healthy aging. Neuroreport 2012;23(8):469–473; doi: 10.1097/WNR.0b013e328353e48a 22495038

[10] Legault I, Allard R, Faubert J. Healthy older observers show equivalent perceptual-cognitive training benefits to young adults for multiple object tracking. Front Psychol 2013;4:323; doi: 10.3389/fpsyg.2013.00323 23761025 PMC3674476

[11] Parsons B, Magill T, Boucher A, et al. Enhancing cognitive function using perceptual-cognitive training. Clin EEG Neurosci 2016;47(1):37–47; doi: 10.1177/1550059414563746 25550444

[12] Spaner CR, Musteata S, Kenny RA, et al. 3-dimensional multiple object tracking training can enhance selective attention, psychomotor speed, and cognitive flexibility in healthy older adults. Ageing Sci Ment Health Stud 2019;3(4):1–12; doi: 10.31038/ASMHS.2019341

[13] Musteata S, Yoshida K, Baranzini D, et al. Perceptual-cognitive training can improve cognition in older adults with subjective cognitive decline. Ageing Sci Ment Health Stud 2019;3(6):1–15; doi: 10.31038/ASMHS.2019361

[14] Snowden T, Ohlhauser L, Mayoh M, et al. Abstracts from The 39th Annual Symposium of the National Neurotrauma Society, including the AANS/CNS joint section on neurotrauma and critical care. Mary Ann Liebert, Inc., publishers: New Rochelle, NY; 2022; p. A-1-A-128; doi: 10.1089/NEU.2022.29126.ABSTRACTS

[15] Corbin-Berrigan LA, Faubert J, Gagnon I. NeuroTracker as a potential mean of active rehabilitation in children with atypical mild traumatic brain injury recovery: a pilot safety study. Transl Sports Med 2020;3(13):235–242; doi: 10.1002/tsm2.132

[16] Lysenko-Martin M, Hutton C, Sparks T, et al. Multiple object tracking scores predict post-concussion status years after mild traumatic brain injury. J Neurotrauma 2020;37(16):1777–1787; doi: 10.1089/neu.2019.6842 31950862

[17] Romeas T, Guldner A, Faubert J. 3D-multiple object tracking training task improves passing decision-making accuracy in soccer players. Psychol Sport Exerc 2016;22:1–9; doi: 10.1016/j.psychsport.2015.06.002

[18] Bernard C. Changing the way we report, interpret, and discuss our results to rebuild trust in our research. eNeuro 2019;6(4):ENEURO.0259-19.2019; doi: 10.1523/ENEURO.0259-19.2019 10.1523/ENEURO.0259-19.2019 PMC6709206 31453315

[19] Cumming G. The new statistics: why and how. Psychol Sci 2014;25(1):7–29; doi: 10.1177/0956797613504966 24220629

[20] Ho J, Tumkaya T, Aryal S, et al. Moving beyond p values: everyday data analysis with estimation plots. 2019;16(7):565–566; doi: 10.1038/s41592-019-0470-3 31217592

[21] Faubert, J. Professional athletes have extraordinary skills for rapidly learning complex and neutral dynamic visual scenes. Sci Rep 2013;3:1154; doi: 10.1038/srep01154 23378899 PMC3560394

[22] Junyent QL, Blázquez PA, Fortó SJ, et al. Perceptual-cognitive training with the Neurotracker 3D-MOT to improve performance in three different sports. Apunts Educación Física y Deportes 2015;119:97–108; doi: 10.5672/APUNTS.2014-0983.ES.(2015/1).119.07

[23] Mangine GT, Hoffman JR, Wells AJ, et al. Visual tracking speed is related to basketball-specific measures of performance in NBA players. J Strength Cond Res 2014;28(9):2406–2414; doi: 10.1519/JSC.0000000000000550 24875429

[24] Vartanian O, Coady L, Blackler K. 3D multiple object tracking boosts working memory span: implications for cognitive training in military populations. Mil Psychol 2016;28(5):353–360; doi: 10.1037/mil0000125

[25] Corbin-Berrigan LA, Kowalski K, Faubert J, et al. Could Neurotracker be used as a clinical marker of recovery following pediatric mild traumatic brain injury? An exploratory study. Brain Inj 2020;34(3):385–389; doi: 10.1080/02699052.2020.1723699 32013583

