# Supplementary material for: A Protocol for Remote Cognitive Training Developed for Use in Clinical Populations During the COVID-19 Pandemic
Source: Neurotrauma Rep. 2023 Aug 14;4(1):522–32. doi: 10.1089/neur.2023.0009 (PMC10460963; doi:10.1089/neur.2023.0009)
Supplement: Supplemental data [file Suppl_FileS1.pdf]

Using the NeuroTrackerX Program at home for participation in a research study.

Visual and Written Instructions

Insert researcher contact information here;
Appendix 1; [insert title of paper here]

Study Timeline and Overview

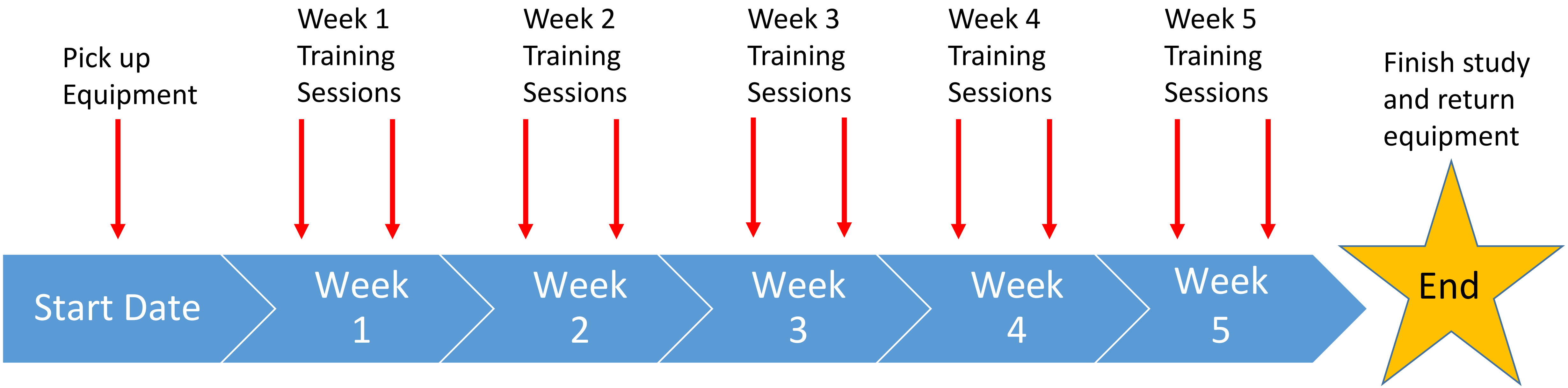

- This study involves 10 training days over 5 weeks (2 training days per week)
- Each training day is made up of 3 training sessions (~6-8 minutes each session, ~20-25 minutes total per day)
- Each training session is made up of 20 short trials of the same task

Navigating NeuroTrackerX

When you open NeuroTrackerX you will see this screen.

1. Enter your user ID that has been provided to you
2. Enter the password that has been provided to you
3. Press the orange LOGIN button

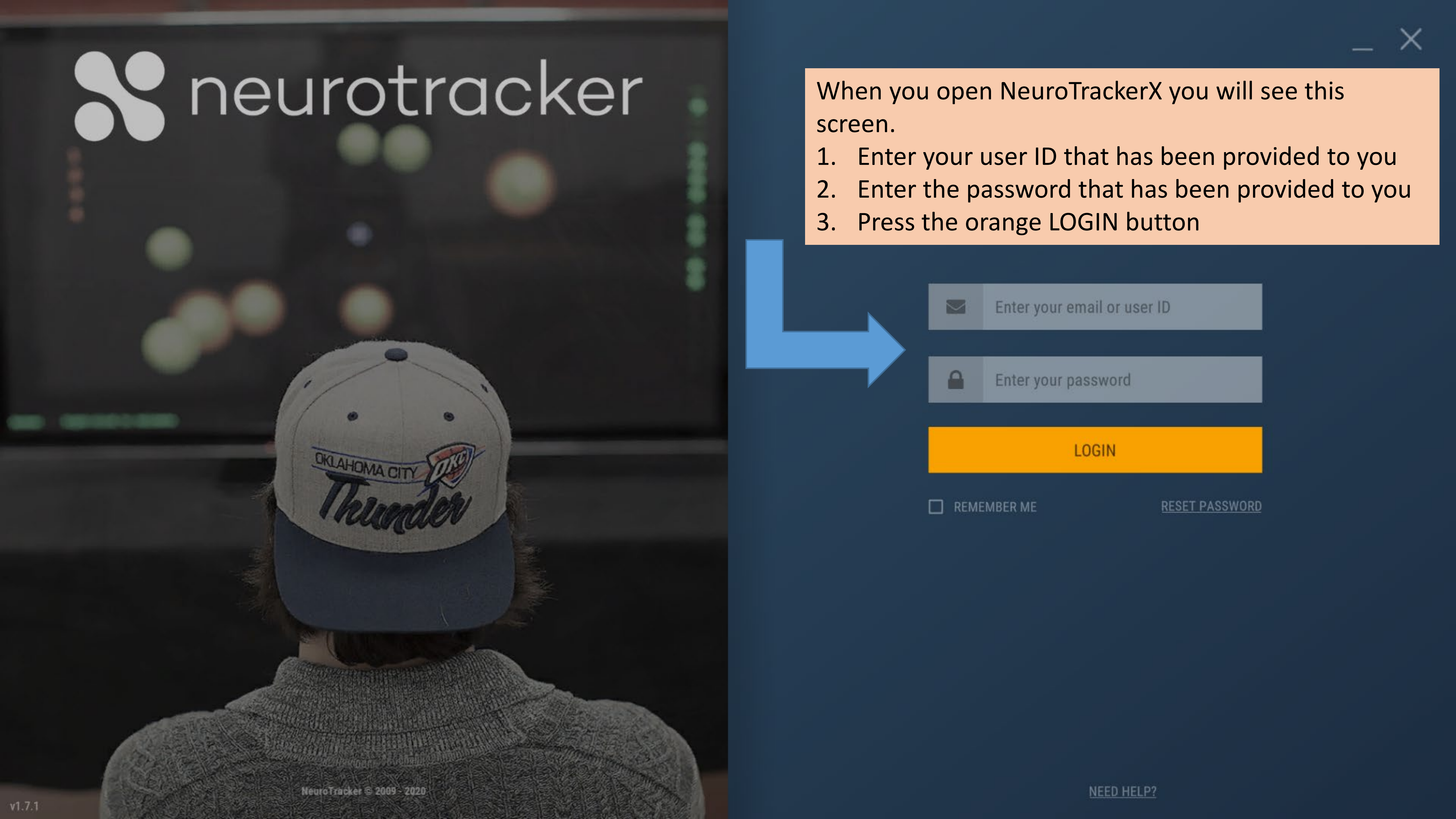

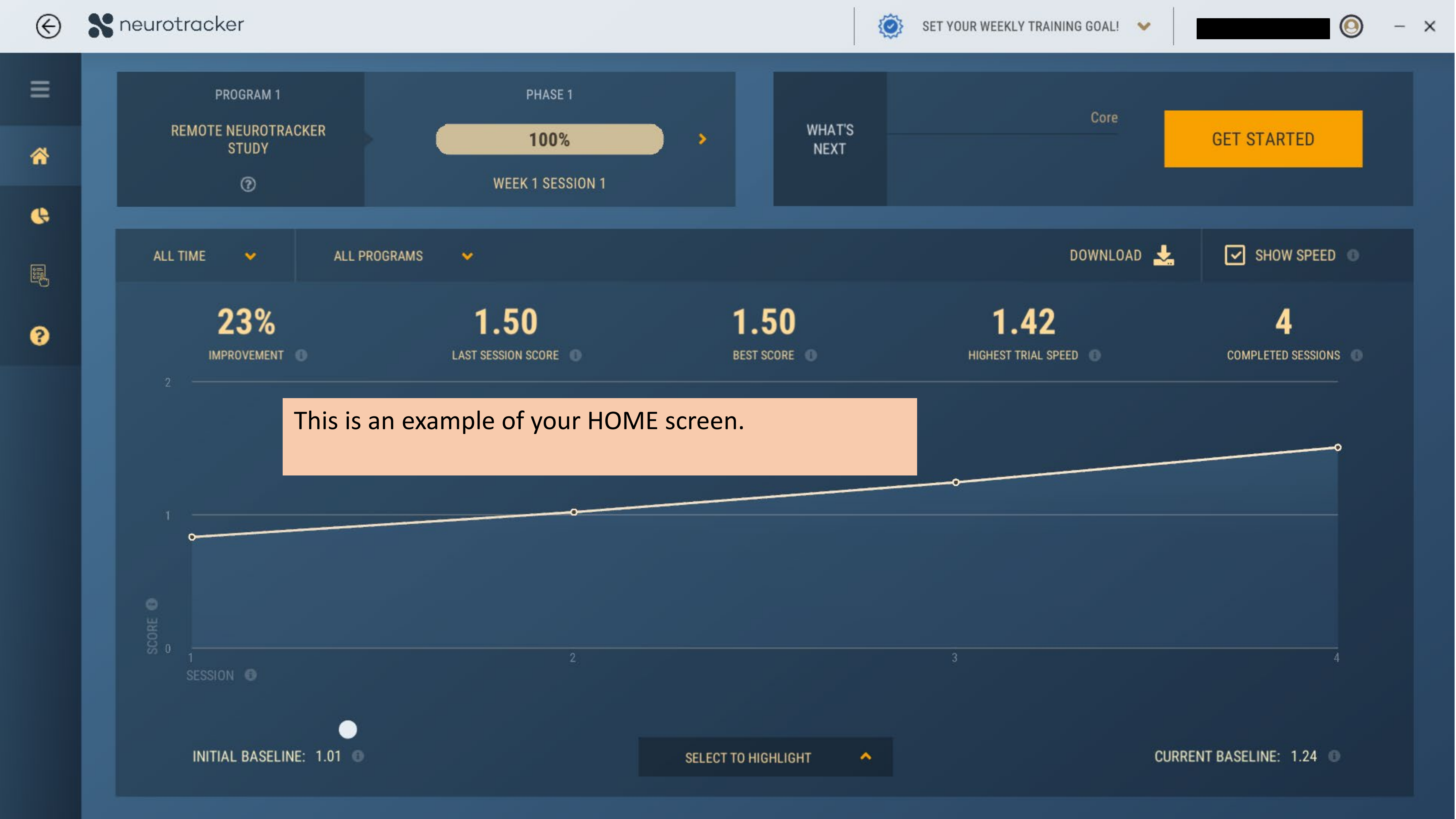

This is an example of your HOME screen.

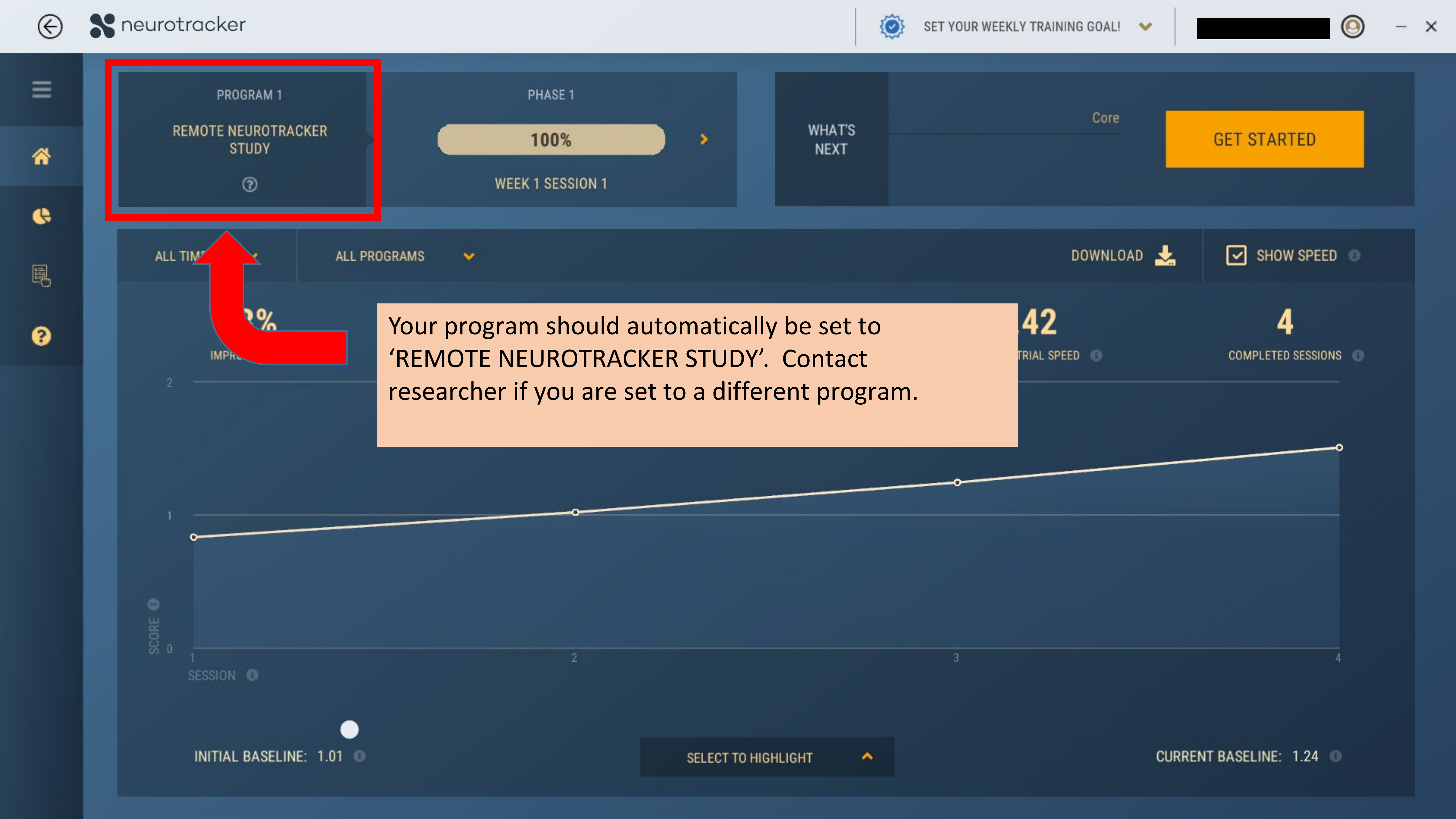

Your program should automatically be set to ‘REMOTE NEUROTRACKER STUDY’. Contact researcher if you are set to a different program.

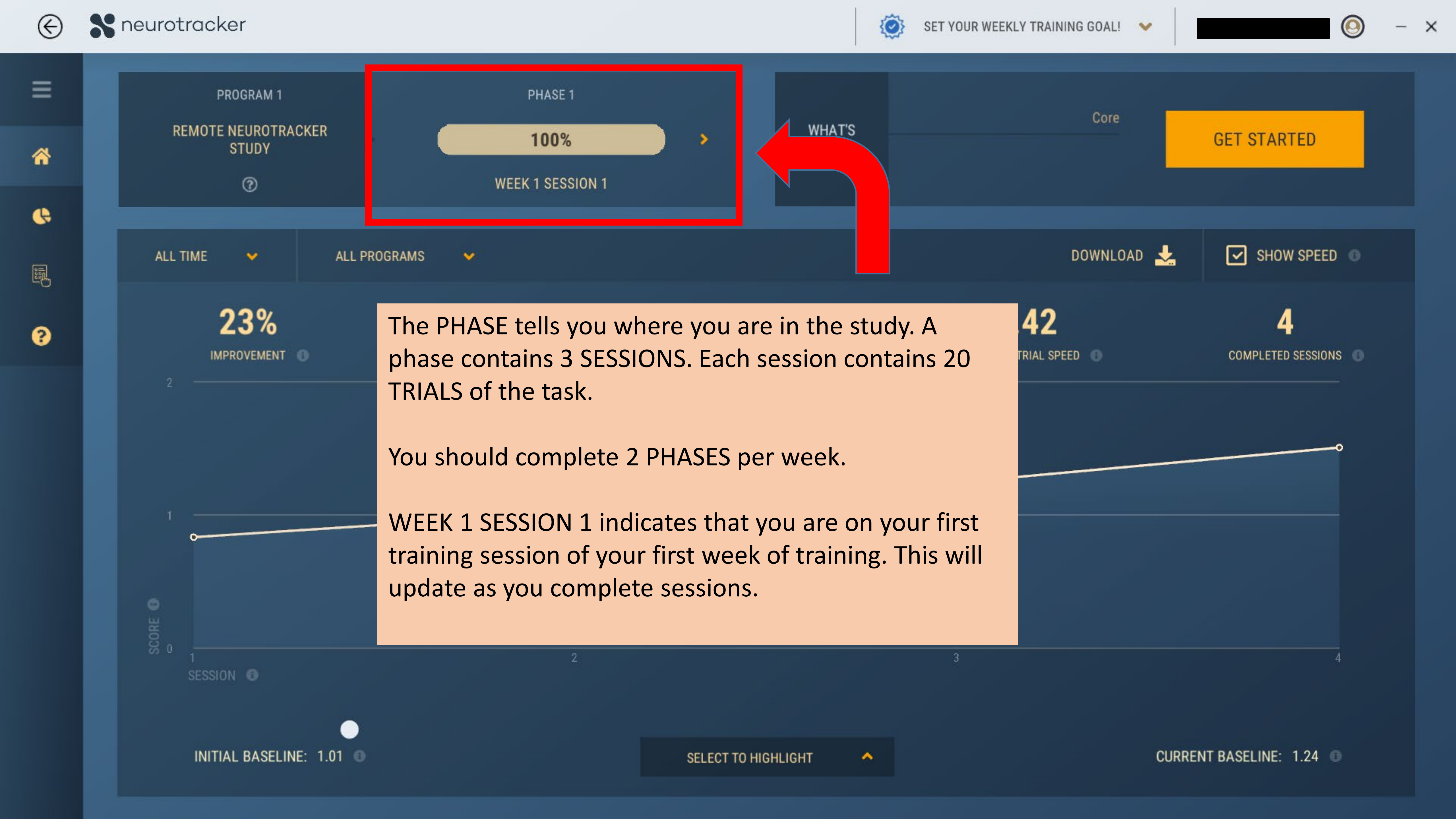

The PHASE tells you where you are in the study. A phase contains 3 SESSIONS. Each session contains 20 TRIALS of the task.

You should complete 2 PHASES per week.

WEEK 1 SESSION 1 indicates that you are on your first training session of your first week of training. This will update as you complete sessions.

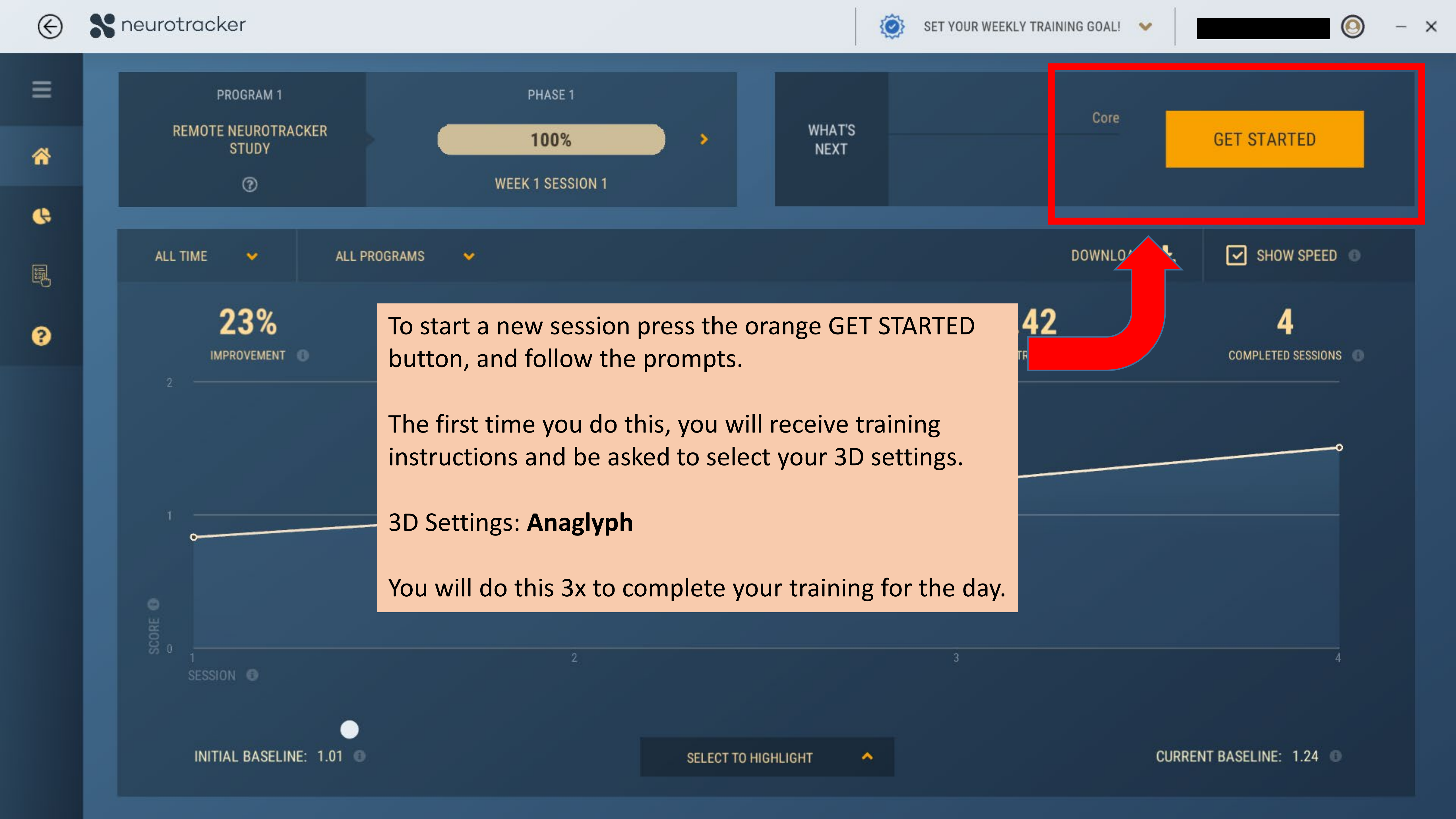

To start a new session press the orange GET STARTED button, and follow the prompts.

The first time you do this, you will receive training instructions and be asked to select your 3D settings.

3D Settings: **Anaglyph**

You will do this 3x to complete your training for the day.

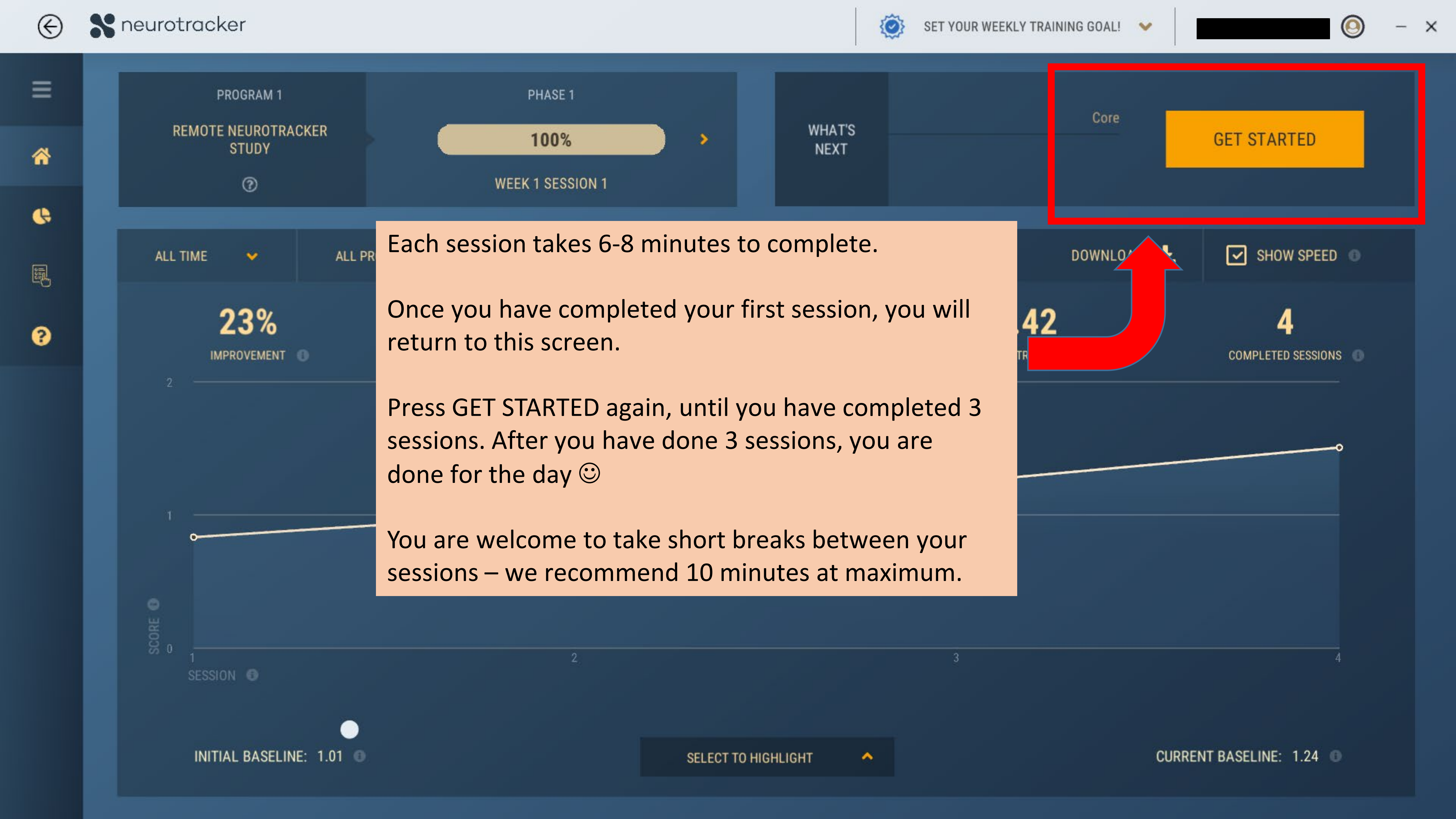

Each session takes 6-8 minutes to complete.

Once you have completed your first session, you will return to this screen.

Press GET STARTED again, until you have completed 3 sessions. After you have done 3 sessions, you are done for the day ☺

You are welcome to take short breaks between your sessions – we recommend 10 minutes at maximum.

This is where you will see your scores on NeuroTracker.

After you have completed 2 PHASES (3 sessions each) you will be able to see percent improvements, scores and a graph of your performance over time.

See later slides for definitions of each phrase used in this section

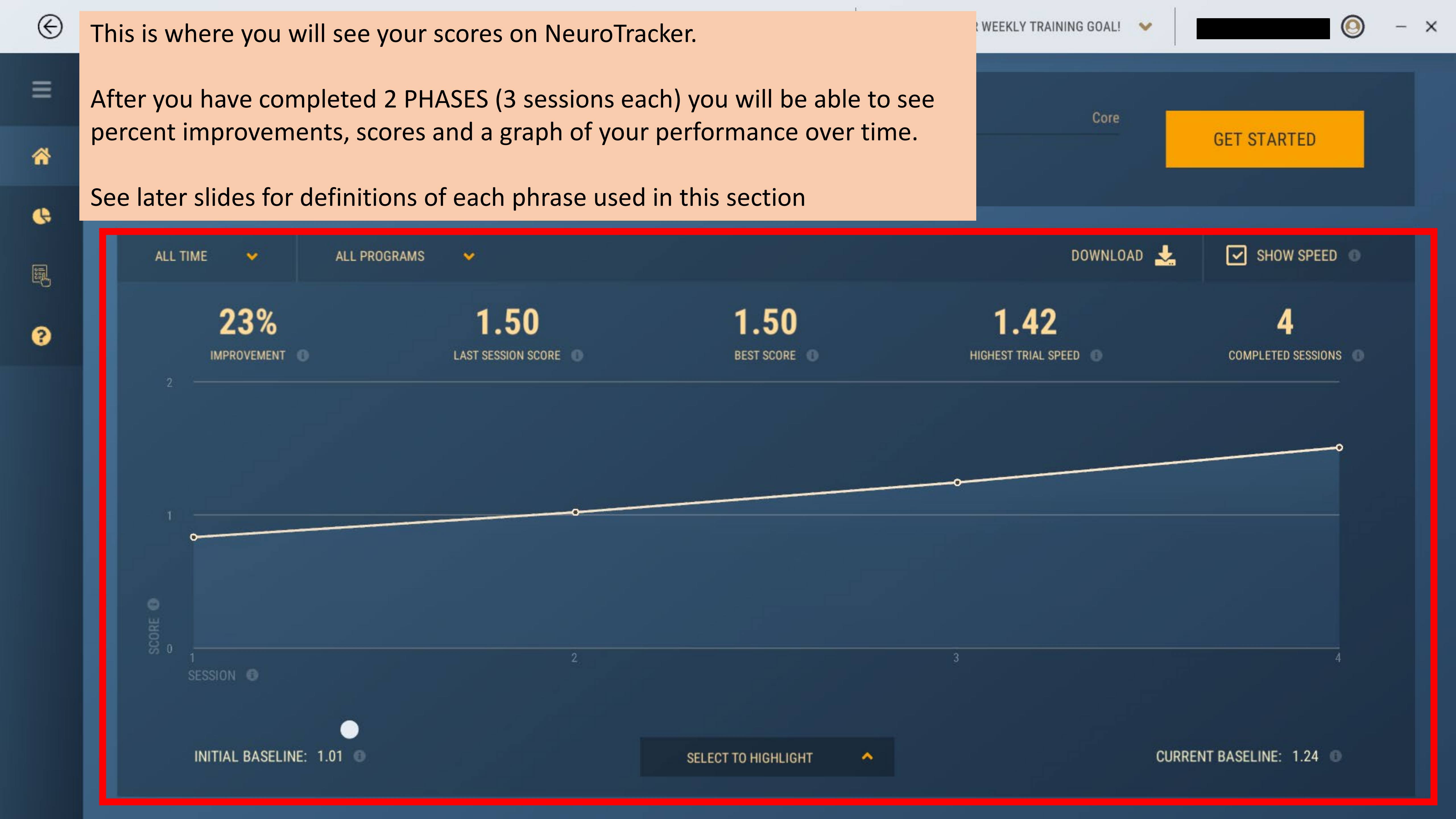

Press the PIE CHART on the left side of the screen for a more in-depth look at your training results. We will go over this together at the end of the study.

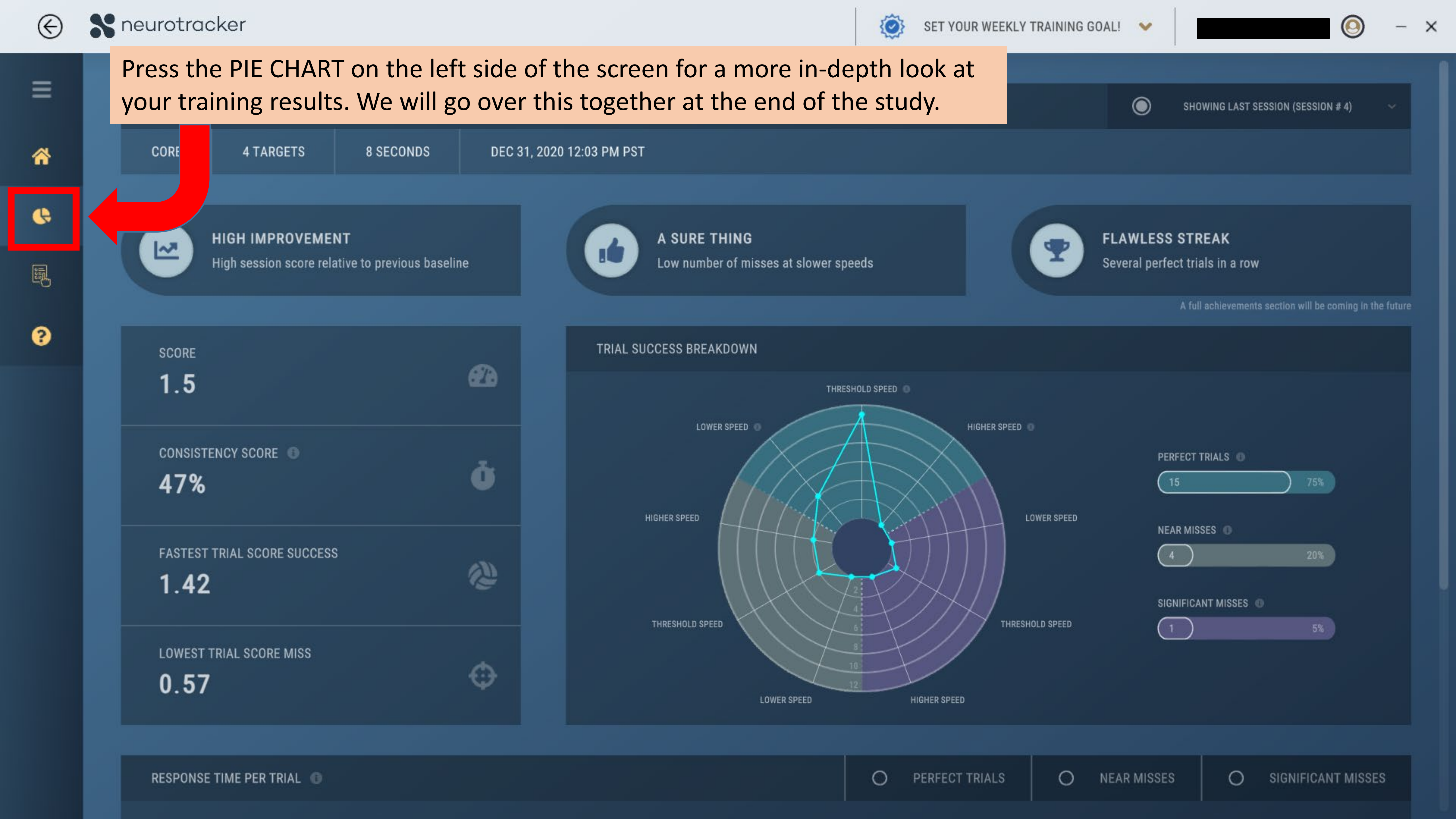

Written Instructions for using NeuroTrackerX the first time.

1. Open NeuroTrackerX and enter your UserID and Password. Both of these will have been emailed to you.
2. A pop-up window will appear with the Terms and Conditions of Use. You must agree to these terms to continue using the program.
3. A pop-up window will appear to explain the training dashboard, program progression and changing profile settings. Click 'NEXT' to move through the explanations.
4. Select 'GET STARTED' in the upper right corner of the screen to begin your first session!
5. Enter your 3D settings (ANAGLYPH)
6. Follow the instructions in the 'HOW TO TRAIN' pop-up, and follow the prompts to begin your session
7. After each session you will see your score, and be able to enter any comments. Feel free to include anything you noticed during your session (I was tired, distracted by my pet, there was construction outside etc.)
8. Congratulations on completing your first session!
9. You need to complete 3 sessions in one sitting. Press 'GET STARTED' again to complete your second session, and again to complete your third session.
10. Once you have finished your 3 sessions, you are done for the day! Be sure to do this 2 times per week (for a total of 6 sessions over 2 days). If you have questions about this please contact the researcher.

TIPS for Using NeuroTrackerX

SETTING-UP

- If you borrowed a computer monitor, see attached document for set up help.
- Your computer monitor should be at a height such that your eyes are level with the middle of the screen. If you are sitting to high or too low, the 3D may be distorted.
- You should sit at a distance equal to the diagonal of your screen size.
  - The 3D may be distorted if you sit too close or too far.
  - Example: If you are using a 24” monitor, sit aprox. 24” away
- You should sit in a quiet area, free from distractions while engaging in NeuroTracker training
  - Turn off music, silence your phone, turn off the TV
  - It is OK to have coffee/tea and snacks with you during the sessions
- We do NOT recommend putting the 3D glasses on prior to your screen being in 3D. This can cause dizziness and headaches. You are welcome to wear the 3D glasses overtop of your regular glasses.

TIPS for Using NeuroTrackerX

<u>During the Sessions:</u>

- To select targets, you can use the number pad on your keyboard, or you can use your mouse to select the targets
- To deselect a target, either type its assigned number again, or click on the target again
- Once you have selected 4 targets, you will not be able to change your answer
- To PAUSE: press the ESC key. Note: the session will only pause once the balls are no longer moving
- To QUIT: press the ESC key and press quit. If you did not finish your session, do not save it. You will have to re-do this session.
- Stare at the centre dot in the middle of the screen, and do your best to use your peripheral vision to follow the targets. This will likely be very challenging at first!
- If it starts out too fast, just do your best and do not fret. Soon the program will learn the perfect speed for you!
- Have fun! You are not expected to get everything right. The system works to challenge you, so you should expect to have a range of successes and failures.

TIPS for Using NeuroTrackerX

<u>Analyzing your results:</u>

- Following completion of all your training sessions, together we will go over your progress throughout the sessions
- On your dashboard (main screen), you will begin to see your scores as you complete sessions
- Your SCORE from each session gives you an idea of your "Speed Threshold". A Speed Threshold is the speed at which you can successfully track the targets about 50% of the time. A higher score means that you can track the targets at a faster rate. The goal of this program is to see how high you can get your score.
- **Initial Baseline**: This is the average score of your first three sessions
- **Current Baseline**: This is the average score of your most recent three sessions (This will only appear once you have done at least 4 sessions)
- **% Improvement**: This compares your current baseline to your initial baseline. Sometimes it will be positive (you scored higher), and sometimes it will be negative (you scored lower).
- **Best Score**: This is your highest session score
- **Highest Trial Speed**: This is your highest trial speed that you have successfully tracked the targets

TIPS for Using NeuroTrackerX

Creating a Training Schedule:

- For this research study, we ask that you complete 10 phases over 5 weeks. Each phase consists of 3 sessions. Each session consists of 20 trials. This means you should be completing 2 phases per week for 5 weeks.
- We recommend doing your phases 2-3 days apart, and try to do them at fairly similar times, in the same environment. You choose the days and times for your training.
- We will be able to track your progress through the study period, if you fall off track we will check in with you and send you friendly reminders ☺

Good Example ☺	Bad Example ☹
• Jane schedules her weekly NeuroTracker training for Monday's and Thursdays ☺<br>• Jane tries to start her Phases between 10-11am each time ☺<br>• Jane takes quick 5 minute breaks between each session to rest ☺<br>• Jane always does her training in a quiet room with few distractions ☺	• John does all his weekly phases back-to-back on the same day ☹<br>• John does his training at random times. Sometimes 7am, and sometimes 11pm ☹<br>• John takes very long, 1 hour breaks between sessions ☹<br>• John leaves the TV and music on, and also tries to cook dinner while doing his sessions ☹

TIPS for Using NeuroTrackerX

Helpful Videos:

- To better understand the task, watch a demo of NeuroTracker before you get started on your training.
  - https://www.youtube.com/watch?v=HAnvc_Il0X0&t=46s
- Completing your first NeuroTrackerX Session
  - https://www.youtube.com/watch?v=LVGrehYXGQ0
- Introducing your user dashboard
  - https://www.youtube.com/watch?v=ZVLJVZUF3OM
