# Supplementary material for: A Protocol for Remote Cognitive Training Developed for Use in Clinical Populations During the COVID-19 Pandemic
Source: Neurotrauma Rep. 2023 Aug 14;4(1):522–32. doi: 10.1089/neur.2023.0009 (PMC10460963; doi:10.1089/neur.2023.0009)
Supplement: Supplemental data [file Suppl_TableS1.docx]

**Table 6. Results from the unconditional means model**

| **AIC** | **BIC** | **logLik** |
| --- | --- | --- |
| 69.39 | 81.13 | -31.69 |
| **Random effects – Formula: ~ 1 \| ID**  **Standard Deviation** | **Intercept** | **Residual** |
|  | 0.3105 | 0.2264 |
| **Fixed effects – Formula: Score ~ 1**  **Intercept** | **Value** | **p value** |
|  | 0.801 | 0.000 |

**Table 6 legend.** Results from the unconditional means model with participant ID as the nested variable and random effect, and NeuroTracker scores as the outcome variable. The intercept (0.081) significantly differs from zero, and additional models are justified. AIC; Akaike information Criterion. BIC; Bayesian Information Criterion. logLik; Log Likelihood.
