# Supplementary material for: A Protocol for Remote Cognitive Training Developed for Use in Clinical Populations During the COVID-19 Pandemic
Source: Neurotrauma Rep. 2023 Aug 14;4(1):522–32. doi: 10.1089/neur.2023.0009 (PMC10460963; doi:10.1089/neur.2023.0009)
Supplement: Supplemental data [file Suppl_TableS2.docx]

**Table 7. Results from the unconditional growth model**

| **AIC** | **BIC** | **logLik** |
| --- | --- | --- |
| -118.19 | -71.23 | 71.09 |
| **Random effects – Formula: ~ 1 \| ID**  **Standard Deviation** | **Intercept** | **Residual** |
|  | 0.3109 | 0.1664 |
| **Fixed effects – Formula: Score ~ Session**  **Intercept** | **Value** | **p value** |
|  | 0.505 | 0.000 |
| **Session 1** | 0.128 | 0.001 |
| **Session 2** | 0.208 | 0.000 |
| **Session 3** | 0.253 | 0.000 |
| **Session 4** | 0.309 | 0.000 |
| **Session 5** | 0.356 | 0.000 |
| **Session 6** | 0.382 | 0.000 |
| **Session 7** | 0.438 | 0.000 |
| **Session 8** | 0.434 | 0.000 |
| **Session 9** | 0.496 | 0.000 |

**Table 7 legend.** Results from the unconditional growth model with a fixed slope, with participant ID as the nested variable and random effect, and Session as a fixed effect. AIC; Akaike information Criterion. BIC; Bayesian Information Criterion. logLik; Log Likelihood.
