# Supplementary material for: A Protocol for Remote Cognitive Training Developed for Use in Clinical Populations During the COVID-19 Pandemic
Source: Neurotrauma Rep. 2023 Aug 14;4(1):522–32. doi: 10.1089/neur.2023.0009 (PMC10460963; doi:10.1089/neur.2023.0009)
Supplement: Supplemental data [file Suppl_TableS3.docx]

**Table 8. Results from the conditional growth model with group as a predictor**

| **AIC** | **BIC** | **logLik** |
| --- | --- | --- |
| -118.37 | -67.50 | 72.18 |
| **Random effects – Formula: ~ 1 \| ID**  **Standard Deviation** | **Intercept** | **Residual** |
|  | 0.3018 | 0.1663 |
| **Fixed effects – Formula: Score ~ Session**  **Intercept** | **Value** | **p value** |
|  | 0.427 | 0.000 |
| **Session 1** | 0.129 | 0.001 |
| **Session 2** | 0.208 | 0.000 |
| **Session 3** | 0.253 | 0.000 |
| **Session 4** | 0.309 | 0.000 |
| **Session 5** | 0.356 | 0.000 |
| **Session 6** | 0.382 | 0.000 |
| **Session 7** | 0.439 | 0.000 |
| **Session 8** | 0.435 | 0.000 |
| **Session 9** | 0.497 | 0.000 |
| **Group** | 0.149 | 0.1489 |

**Table 8 legend.** Results from the conditional growth model with a fixed slope. The model included participant ID as the nested variable and random effect, Session as a fixed effect, and Group as a predictor. Group was not a significant predictor of NeuroTracker score by Session. AIC; Akaike information Criterion. BIC; Bayesian Information Criterion. logLik; Log Likelihood.
