# Supplementary material for: A Protocol for Remote Cognitive Training Developed for Use in Clinical Populations During the COVID-19 Pandemic
Source: Neurotrauma Rep. 2023 Aug 14;4(1):522–32. doi: 10.1089/neur.2023.0009 (PMC10460963; doi:10.1089/neur.2023.0009)
Supplement: Supplemental data [file Suppl_TableS4.docx]

**Table 9. Equivalent improvement in NeuroTracker score from first to final sessions**.

| Comparison | Paired mean difference  At-home; In-lab | 95% CI  At-home; In-Lab | Test value, *p* value (paired Student’s t-test) At-home; In-lab | Interpretation |
| --- | --- | --- | --- | --- |
| First to final  (1; 10) | 0.494; 0.463 | [0.315, 0.761];  [0.237, 0.694] | *t*[27] = -4.6376, *p* = 0.0000747;  *t*[22] = -4.4937, *p* = 0.000174 | No difference |

**Table 9 Legend.** Mean-difference was calculated as (in-lab score minus at-home score) with 95% CIs. CIs were calculated using 5000 bootstrap samples, and are bias-corrected and accelerated. For each permutation *p* value, 5000 reshuffles of the at-home and in-lab labels were performed.
